# Detection and characterization of traumatic bile leaks using Gd-EOB-DTPA enhanced magnetic resonance cholangiography

**DOI:** 10.1038/s41598-018-32976-0

**Published:** 2018-10-02

**Authors:** Yon-Cheong Wong, Li-Jen Wang, Cheng-Hsien Wu, Huan-Wu Chen, Chen-Ju Fu, Kuo-Ching Yuan, Being-Chuan Lin, Yu-Pao Hsu, Shih-Ching Kang

**Affiliations:** 1 Emergency and Critical Care Radiology, Department of Medical Imaging and Intervention, Chang Gung Memorial Hospital, Chang Gung University, Taoyuan City, Taiwan; 2 Division of Trauma and Emergency Surgery, Department of Surgery, Chang Gung Memorial Hospital, Chang Gung University, Taoyuan City, Taiwan; 3 Center for Advanced Molecular Imaging and Translation, Taoyuan City, Taiwan

## Abstract

Expanding bile leaks after blunt liver trauma require more aggressive treatment than contained bile leaks. In this retrospective study approved by institution review board, we analyzed if non-invasive contrast-enhanced magnetic resonance cholangiography (CEMRC) using hepatocyte-specific contrast agent (gadoxetic acid disodium) could detect and characterize traumatic bile leaks. Between March 2012 and December 2014, written informed consents from 22 included patients (17 men, 5 women) with a median age of 24.5 years (IQR 21.8, 36.0 years) were obtained. Biliary tree visualization and bile leak detection on CEMRC acquired at 10, 20, 30, 90 minutes time points were independently graded by three radiologists on a 5-point Likert scale. Intraclass Correlation (ICC) was computed as estimates of interrater reliability. Accuracy was measured by area under receiver operating characteristic curves (AUROC). Biliary tree visualization was the best on CEMRC at 90 minutes (score 4.30) with excellent inter-rater reliability (ICC = 0.930). Of 22 CEMRC, 15 had bile leak (8 expanding, 7 contained). The largest AUROC of bile leak detection by three radiologists were 0.824, 0.914, 0.929 respectively on CEMRC at 90 minutes with ICC of 0.816. In conclusion, bile leaks of blunt liver trauma can be accurately detected and characterized on CEMRC.

## Introduction

Biliary injuries can occur at the time of blunt liver trauma^1–5^. Its frequency has been reported to range from 3% in low grade liver trauma to about 15% in high grade trauma^1,3–10^. However, the frequency may have been underestimated because biliary injuries are initially asymptomatic and manifestation of symptoms is always delayed for weeks until biliary injuries are complicated with superinfection or biliary tree obstruction^3,5^. Expanding bile leaks associated with liver capsular rupture require more aggressive treatment as compared to contained bile leaks, similar to liver injuries with extracapsular vascular extravasation^11^. Therefore, complicated biliary injuries often require further diagnostic work up as well as complex therapeutic surgical or endoscopic treatment procedures^4–7,12–14^. These result in a higher medical cost, prolonged hospital stay or higher mortality rates in more severe cases^3,13,14^.

Hepatobiliary iminodiacetic acid scan can serve as a test for bile leaks detection^15,16^. However, the lack of anatomical details on planar images can sometimes misinterpret radioactive tracer in duodenum or normal anatomical variant of biliary tree as bile leaks^15^. Delayed imaging up to twenty four hours is required for biloma confirmation and that is not practical among trauma patients^15^. The site of biliary injuries and extent of bile leak can be directly evaluated with endoscopic retrograde cholangiography and percutaneous transhepatic cholangiography^3^. These diagnostic procedures are standard reference for biliary injuries and can also provide therapeutic intervention^7^. In addition, intrahepatic fluid accumulation can be aspirated and analyzed for bile content, or it can be contrasted by percutaneous cavitography for evaluation if the intrahepatic fluid communicates with biliary tree^17,18^. However, all these invasive procedures can pose additional risks to recently injured patients. Therefore, they are rarely the first-line diagnostic procedure for a suspected bile leak.

With the advent of magnetic resonance imaging (MRI) and introduction of hepatocyte specific contrast agents, MRI has revolutionize hepatobiliary imaging. About 50% of the intravenously injected hepatocyte-specific MRI contrast agent such as gadoxetic acid disodium is excreted by hepatocytes into biliary system shortening the T1 effect of bile on MRI^19,20^. Extravasation of this biliary contrast medium from biliary tree to the intrahepatic or perihepatic biloma is readily detected and easily differentiated from other fluid collections^21^. Consequently, the gadoxetic acid disodium contrast-enhanced magnetic resonance cholangiography (CEMRC) can be used to evaluate hepatic excretory function and anatomy of biliary tree^19–22^. However, most of the published reports on CEMRC are focused on liver tumors and post-operative bile leak^20,23^. Little has been mentioned about its acquisition time and efficacy for blunt biliary injuries detection.

Therefore, optimization of CEMRC for detecting and characterizing traumatic bile leaks was performed in this study. Our purpose is to analyze the diagnostic performance of CEMRC for traumatic bile leak and to compare different characteristics of bile leak (expanding or contained) with clinical parameters, specific treatment and complication.

## Results

### Trauma characteristics of patients

A total of 22 patients were included in this study. All had recent major blunt liver trauma ≥grade III injuries according to scoring scheme of American Association for the Surgery of Trauma (AAST). The initial contrast-enhanced CT showed focal traumatic distortion of liver architecture. Three patients had other associated abdominal organs trauma such as spleen (n = 2), kidney (n = 1), duodenum (n = 1) and pancreas (n = 1). The overall median injury severity score was 29 (IQR 17.0, 34.5).

### Final decision and characterization of bile leak

Of 22 patients, 15 (68.2%) patients were determined to have bile leaks at expert-panel consensus decision. Among 15 of them, 7 were type I and 8 were type II bile leaks on CEMRC. Type I bile leak was confined, type II bile leak was expanding in the presence of liver capsule disruption. Additional examinations such as endoscopic retrograde cholangiography (n = 3), percutaneous cavitography and bile stained aspirates (n = 5) were available in this group. Of the other 7 patients who were determined to have no bile leak at expert-panel consensus decision, one was checked on by laparotomy to exclude biliary injury, two were confirmed by a negative result on percutaneous cavitography and aspirates analysis. Four others who recovered rapidly were clinically followed up. They did not develop any biliary complication and were cleared of biliary injury at panel decision.

### Biliary tree visualization on CEMRC

**Figure 1:**
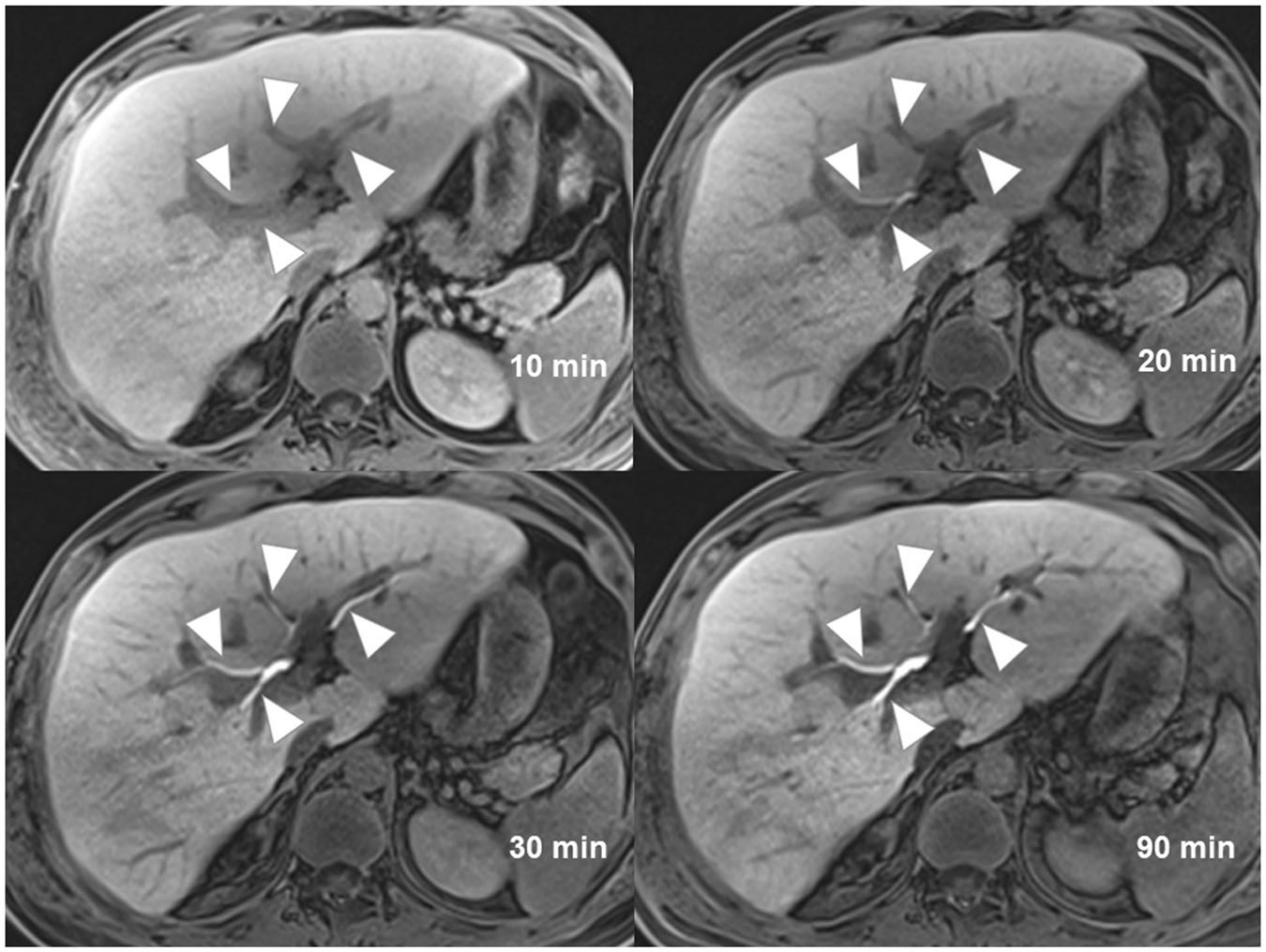
A 36-year-old man with liver injury (not shown). Images of contrast enhanced MR cholangiography acquired at 10 minutes, 20 minutes, 30 minutes and 90 minutes show progressive improvement of intrahepatic bile ducts visualization (arrowheads) from very poor visualization at 10 minutes to excellent visualization at 90 minutes. The average scores given by three readers are 1.00 (10 minutes), 4.00 (20 minutes), 4.67 (30 minutes), 5.00 (90 minutes).

In Table 1, the mean score of biliary tree visualization on CEMRC acquired at 10, 20, 30 and 90 minutes were 2.96, 4.17, 4.29 and 4.30 respectively. The inter-rater reliability was good to excellent with ICC of 0.921, 0.787, 0.717 and 0.930 on CEMRC acquired at 10, 20, 30 and 90 minutes respectively. The best biliary tree visualization was scored on CEMRC acquired at 90 minutes (Fig. 1) and this was substantiated by the best inter-rater reliability test. On the contrary, the poorest biliary tree visualization was scored on CEMRC acquired at 10 minutes by all readers, and it was also supported by an excellent ICC.

**Table 1:** Tabulation of biliary tree visualization presented as mean score ± standard deviation on contrast-enhanced magnetic resonance cholangiography (CEMRC) of different acquisition times graded by three radiologists and the respective intraclass correlation coefficients (ICC).

CEMRC acquisition times	Mean score ± SD	ICC	95% confidence interval of ICC
10 minutes	2.96 ± 1.41	0.921	0.820, 0.966
20 minutes	4.17 ± 0.89	0.787	0.485, 0.912
30 minutes	4.29 ± 0.76	0.717	0.405, 0.875
90 minutes	4.30 ± 0.98	0.930	0.845, 0.970

### Bile leak on CEMRC

The median time to perform CEMRC after blunt liver trauma for the group with a positive result of bile leak did not differ significantly from the group with a negative result (11th day versus 8th day, p = 0.750), as well as for the group with type I bile leak from the group with type II bile leak (10th versus 12th day, p = 0.221).

**Figure 2:**
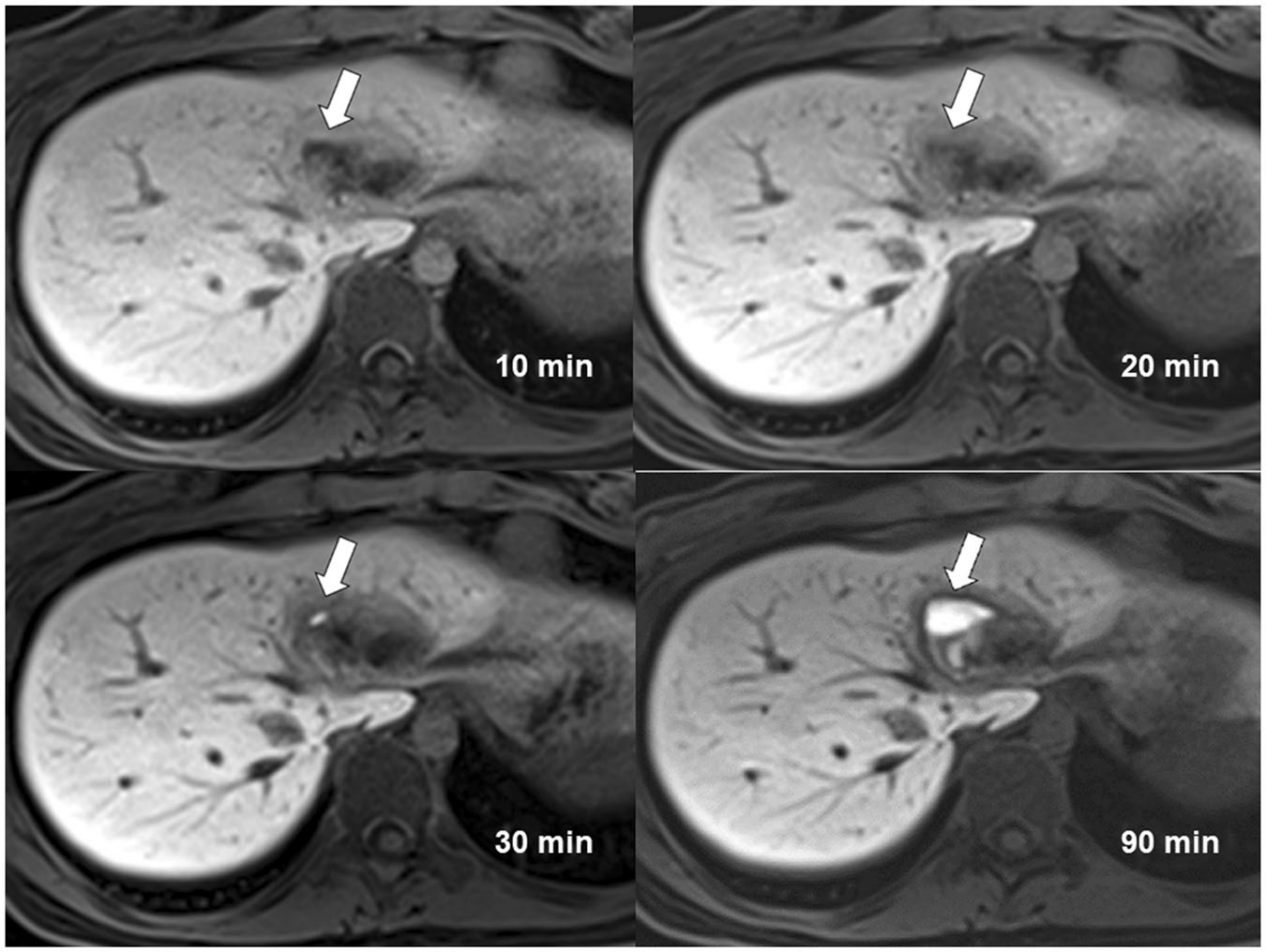
Contrast enhanced MR cholangiography of a 20-year-old woman with a liver injury acquired at 10 minutes, 20 minutes, 30 minutes and 90 minutes. Type I bile leak confined within liver parenchyma is most obviously detected at 90 minutes acquisition (arrow). The average scores given by three readers are 1.33 (10 minutes), 2.00 (20 minutes), 2.00 (30 minutes), 5.00 (90 minutes).

The scores on bile leak detection among three readers increased from CEMRC acquired at 10 minutes to CEMRC acquired at 90 minutes (Fig. 2). The AUROC of the three readers for bile leak detection on CEMRC of different acquisition time points tabulated in Table 2 also increased significantly from 10 minutes to 90 minutes. The AUROC acquired at 90 minutes by three readers were 0.824, 0.914 and 0.929 respectively and the inter-rater reliability was very good with an ICC of 0.816. Although the ICC was higher (0.821 versus 0.816) for CEMRC acquired at 30 minutes than that acquired at 90 minutes, the AUROC for bile leak detection by the three readers on CEMRC acquired at 30 minutes were not only small but also statistically nonsignificant.

**Table 2:** Area under receiver operating characteristic curve (AUROC) for detecting bile leak on contrast-enhanced magnetic resonance cholangiography (CEMRC) of different acquisition times by three radiologists and the respective intraclass correlation coefficients (ICC).

CEMRC acquisition times	AUROC (95% CI) Radiologist A	AUROC(95% CI) Radiologist B	AUROC (95% CI) Radiologist C	ICC (95% CI)
10 minutes	0.514 (0.264, 0.764)	0.562 (0.314, 0.810)	0.624 (0.380, 0.867)	0.533 (0.117, 0.782)
20 minutes	0.724 (0.509, 0.938)	0.714 (0.499, 0.930)	0.781 (0.589, 0.973)	0.734 (0.469, 0.880)
30 minutes	0.600 (0.365, 0.835)	0.743 (0.514, 0.972)	0.695 (0.456, 0.935)	0.821 (0.641, 0.920)
90 minutes	0.824 (0.609, 1.000)	0.914 (0.793, 1.000)	0.924 (0.807, 1.000)	0.816 (0.628, 0.918)

### Comparisons of clinical parameters, specific treatments for biliary injuries, biliary-related complications and length of hospital stay between groups

As summarized in Table 3, patients with bile leak were significantly associated with active liver hemorrhage on admission CT (p = 0.007) and liver angioembolization (p = 0.014) as the initial hemostasis treatment for liver trauma. Data of liver enzymes and bilirubin on arrival as well as length of hospital stay between the groups with and without bile leak did not differ (Table 4). However, the median bilirubin level on arrival were significantly higher (1.80 versus 0.95 mg/dL, p = 0.022) and median length of hospital stay was significantly longer (23 versus 11 days, p = 0.003) respectively in the group with type II bile leak than in the groups with type I bile leak.

**Table 3:** Comparisons of sex, liver injury grades, admission CT with active liver hemorrhage, initial treatment for liver injury, and specific treatment for biliary injuries between groups with and without bile leak as well as between groups with type I and type II bile leak.

Variables	Bile leak		p-value (Fisher’s exact test	Bile leak types		p-value (Fisher’s exact test
	yes (n = 15)	no (n = 7)		Type I (n = 7)	Type II (n = 8)	
Sex			0.135			1.000
women	5	0		2	3	
men	10	7		5	5	
Injury grades			1.000			0.119
III	5	3		4	1	
IV	10	4		3	7	
Active liver hemorrhage on CT			0.007			0.569
no	3	6		2	1	
yes	12	1		5	7	
Initial treatment for liver trauma			0.014			0.200
observation	2	5		2	0	
embolization	13	2		5	8	
Treatment for biliary injuries			0.193			0.041
observation	8	6		6	2	
drainage	7	1		1	6

**Table 4:** Comparisons of age, contrast-enhanced magnetic resonance cholangiography (CEMRC) performed days after trauma, length of hospital stay, aspartate aminotransferase (AST), alanine transaminase (ALT) and bilirubin on arrival between groups with and without bile leak as well as between groups with type I and type II bile leak.

Variables	Bile leak		p-value (Mann-Whitney test)	Bile leak types		p-value (Mann-Whitney test)
	yes (n = 15)	no (n = 7)		Type I (n = 7)	Type II (n = 8)	
Age (years)	24 (21, 32)	35 (22, 38)	0.458	24 (21, 32)	24 (22, 35)	0.772
CEMRC after trauma (days)	11 (9, 13)	8 (8, 22)	0.750	10 (5, 13)	12 (10, 14)	0.221
Length of stay (days)	16 (11, 23)	12 (9, 29)	0.596	11 (9, 16)	23 (20, 32)	0.003
AST (U/L)	317 (203, 649)	414 (186, 460)	0.805	303 (203, 412)	580 (218, 837)	0.105
ALT (U/L)	394 (222, 494)	260 (193, 310)	0.275	357 (245, 459)	471 (140, 820)	0.355
Total bilirubin (mg/dL)	1.10 (0.95, 1.80)	1.50 (0.85, 2.33)	0.826	0.95 (0.73, 1.23)	1.80 (1.10, 2.90)	0.022

Data are presented as median (interquartile range 25%, 75%).

Among 22 patients, 8 underwent catheter drainage, 14 were treated with observation. The eight catheter drainage procedures included three endoscopic retrograde biliary drainage and five percutaneous catheter drainage. Of these 8 patients, seven (7/8, 87.5%) had bile leak. The other one patient who underwent catheter drainage did not have bile leak and the aspirate yielded old blood. Of the 14 patients treated with observation, eight (8/14, 57.1%) had bile leak.

**Figure 3:**
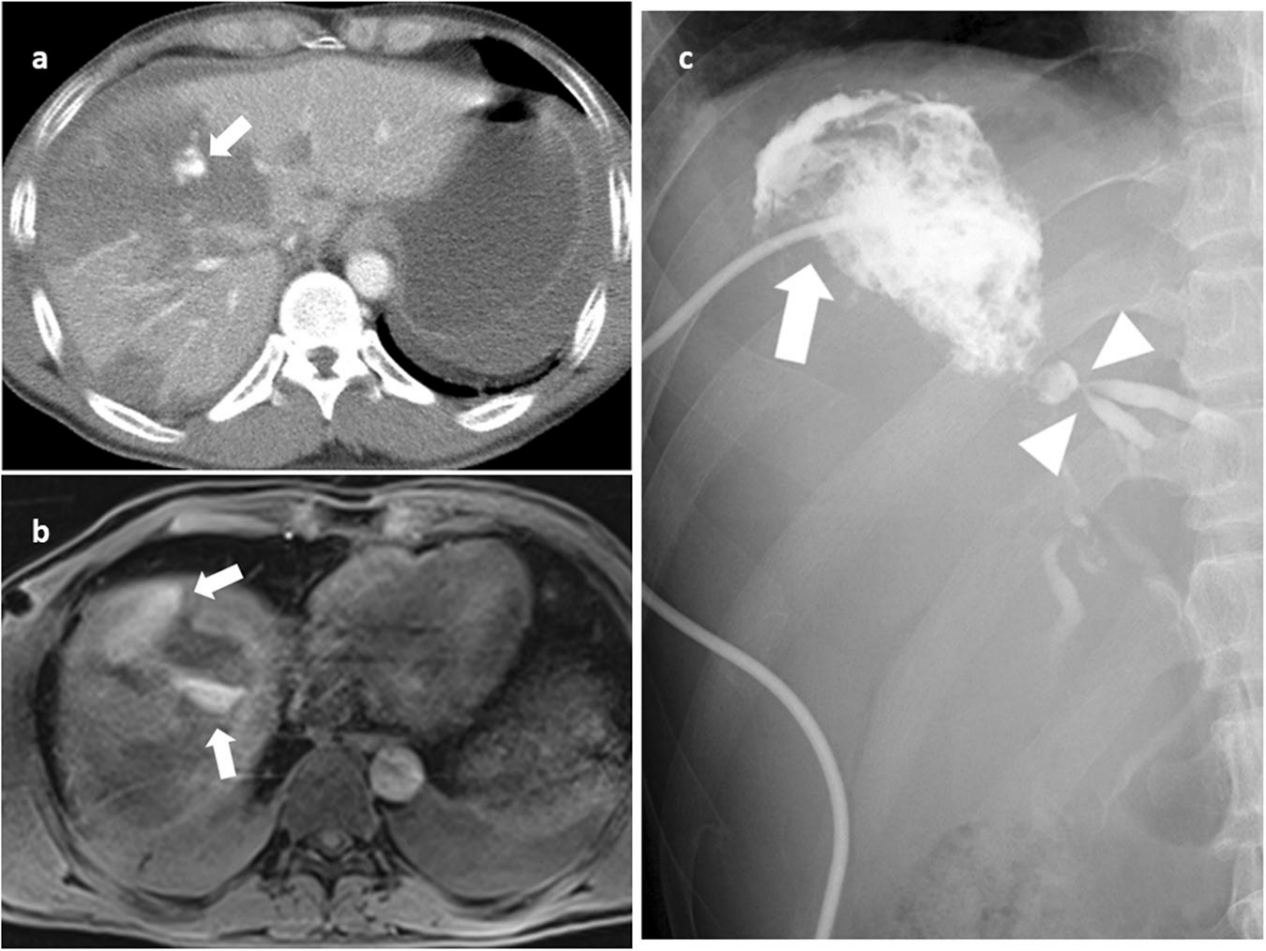
CT, MRI and cavitrography of a 36-year-old man with grade IV liver injury. (**a**) Contrast-enhanced CT in axial plane shows active extravasation of contrast medium (arrow) at the liver parenchyma, treated initially with angioembolization (not shown). (**b**) Contrast enhanced MR cholangiography acquired at 90 minutes in axial plane shows a type II bile leak (arrows) expanding through the disrupted anterior liver capsule to perihepatic space. (**c**) Percutaneous cavitography through a pigtail catheter (arrow) shows a direct communication of the injected iodinated contrast medium (biloma) with the injured intrahepatic bile ducts (arrowheads). He was treated with both percutaneous catheter drainage and endoscopic retrograde biliary drainage and later developed intrahepatic bile duct stricture.

The difference of treatment choice between groups with and without bile leak was not significant. However, as shown in Table 3, the difference of catheter drainage between type I and type II bile leak groups was statistically significant (p = 0.041). One (1/7, 14.3%) patient with type I bile leak and six (6/8, 75.0%) patients with type II bile leak required catheter drainage (see Supplementary Fig. S1). One patient of type II bile leak who was treated with percutaneous and endoscopic retrograde biliary drainage developed severe biliary stricture (Fig. 3). Other patients recovered uneventfully after bile leaks were resolved. None of the 7 patients of the group without bile leak developed jaundice, cholangitis, biliary tree stricture or progressive enlargement of intra-abdominal fluid (intrahepatic or intraperitoneal).

## Discussion

With the advent of magnetic resonance imaging, the diagnostic performance of T2-weighted magnetic resonance cholangiography which capitalizes on the long T2-relaxation property of bile is comparable to that of endoscopic retrograde cholangiography for biliary cancer, biliary stone and biliary anomaly^24^. However, other fluid collections of the liver are difficult to be differentiated from bile leak because all are hyperintense on T2-weighted images^21^.

On the contrary, by using gadoxetic acid disodium as the contrast agent, CEMRC can easily identify the T1-shortening bile leak. It has been reported that CEMRC acquired 20 minutes after administration of Gd-EOB-DTPA is sufficient for obtaining hepatobiliary phase images^25–27^ although the acquisition can range from 20 to 180 minutes^25^. The best acquisition time of CEMRC using Gd-EOB-DTPA among trauma patients has never been investigated. Our main concern in this study is to optimize the acquisition time and expedite the examination for trauma patients. We found that CEMRC of 30-minute and 90-minutes acquisitions have higher scores of biliary tree visualization on a 5-point scale, as compared to CEMRC of 10-minute and 20-minute acquisitions. Furthermore, the reliability test of visualization scores among three radiologists was the best on CEMRC of 90-minute acquisition.

Our study showed that bile leak was also best detected on CEMRC acquired at 90-minute with the highest AUROC. The diagnostic yield for bile leak is better when the biliary tree is more adequately filled up with excreted contrast medium. Similarly, depiction of bile leak was also the best reliable with a very good ICC on CEMRC acquired at 90-minutes among three radiologists.

Not only a high risk of biliary injuries can occur in patients with major liver trauma^14,28^, our results showed that bile leak was significantly associated with active liver hemorrhage on arrival CT. The severe lacerations of major liver trauma not only injure the hepatic vessels, its shearing force can also disrupt bile ducts and portal veins at the portal triad. Active arterial bleeding or pseudoaneurysm is often treated by angioembolization^1,29–31^ for patients who are transiently responsive to fluid resuscitation. We have found that angioembolization is associated with bile leak. This is because angioembolization may compromise the vascular supply to the walls of bile ducts, causing a possibility of local epithelial ischemia and delayed healing at the site of biliary injuries, therefore resulting in delayed bile leak^3,14,28^.

Traumatic biliary injuries with type I bile leak can resolve spontaneously. In contrast, the type II bile leak is associated with a higher level of total serum bilirubin and catheter drainage. Even though one patient with expanding bile leak underwent endoscopic retrograde biliary stenting and percutaneous transhepatic biliary drainage, he still developed biliary stricture. Moreover, patients with type II bile leak usually have a longer length of hospital stay than those with type I bile leak.

Biliary injury is often underestimated because the diagnostic tools are too invasive for asymptomatic patients. We have proven that non-invasive CEMRC is feasible to detect and characterize bile leak in trauma patients. However, good quality CEMRC depends on liver excretion function of Gd-EOB-DTPA^21,25^ which is mediated by the same transporter system of bilirubin transport.

In this study, we only included patients with major blunt liver trauma who were co-operative and compliant with the MRI safety requirements. The implanted cardiac devices or embedded metallic foreign bodies after trauma can prohibit patients from undergoing CEMRC. Small sample size is therefore the main limitation of this study. The second limitation is that we categorized patients into groups with and without bile leak by expert-panel consensus because verification by the ideal endoscopic retrograde cholangiography is too invasive for asymptomatic patients. The third limitation is that we did not have patients with very high serum level of bilirubin. The total bilirubin level of our patients ranged from 0.50 to 3.60 mg/dL. We do not know whether CEMRC acquired at 90-minute is still optimal for bile leak detection if bilirubin level exceeds 3.60 mg/dL. The fourth limitation is that we did not acquire CEMRC at longer than 90 minutes time points because MRI scheduling is always tight and we cannot afford to decrease MRI throughput per day. Fortunately, excretion of Gd-EOB-DTPA can be visualized on CEMRC acquired at 90-minutes in all patients in this study. We did not investigate whether or not CEMRC at 60 minutes time point would have yielded a similar results as 90 minutes. This has left us an opportunity for clarification in the next study.

In conclusion, CEMRC is feasible to provide both anatomical and functional information for traumatic bile leaks among major liver trauma patients with a history of active liver bleeder treated by angioembolization. Bile leak is most optimally detected on CEMRC acquired at 90 minutes time point after intravenous injection of Gd-EOB-DTPA. Blunt liver trauma patients with type II (expanding) bile leak are associated with higher level of total serum bilirubin, more likely to undergo catheter drainage and a longer length of hospital stay than patients with type I (contained) bile leak.

## Methods

### Subjects

**Figure 4:**
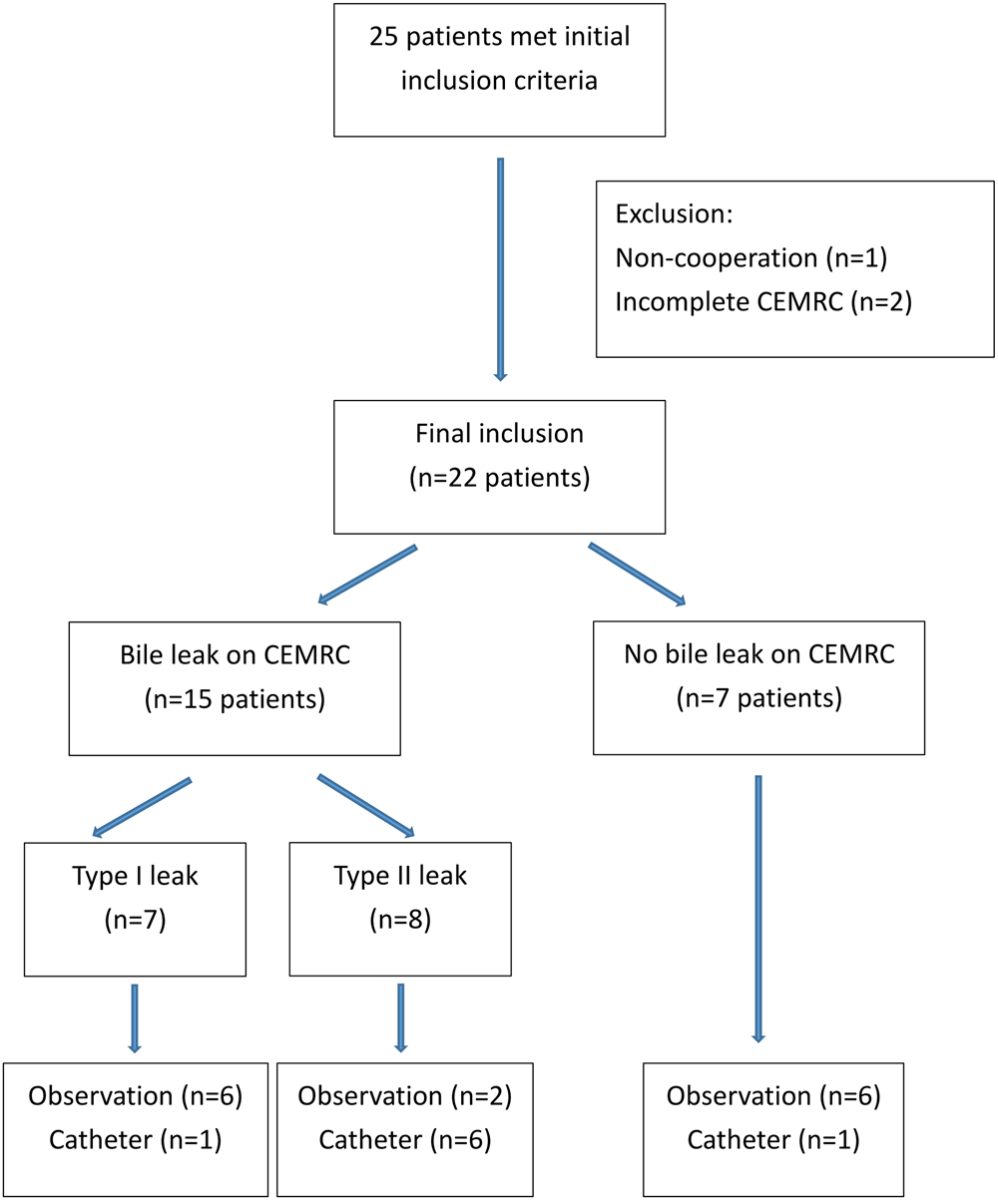
Flowchart of patient inclusion. It shows the selection of major liver trauma patients for the study. CEMRC = gadoxetic acid disodium contrast enhanced magnetic resonance cholangiography.

This retrospective study on CEMRC for biliary injuries was approved by Chang Gung Memorial Hospital’s institution review board and all methods were performed in accordance with the relevant guidelines (RE: 99–3856B). A written informed consent was obtained from every participant. From March 2012 to December 2014, a total of 25 consecutive patients met the inclusion criteria (adults ≥20 years old, clear consciousness without intubation, compliant with the MRI safety requirements, recent major blunt liver trauma ≥grade III injuries according to AAST scoring scheme, and status post stabilized by non-operative management). Among them (Fig. 4), one was excluded because of poor cooperation during scanning, two were excluded because of incomplete CEMRC examination. Finally, 22 patients with a median age of 24.5 years (IQR 21.8, 36.0 years) including 17 men with a median age of 24.0 years (IQR 21.5, 36.0 years) and 5 women with median age of 25.0 years (IQR 21.5, 47.5 years) were included. A review of the trauma registry documented that their median injury severity score was 29 (IQR 17.0, 34.5). They constituted 12.4% of patients with ≥grade III blunt liver trauma.

### Magnetic resonance imaging technique

The CEMRC was performed on a median of 10.5 days (IQR 8.0, 15.5 days) after blunt liver trauma at the time when the patients had been evaluated by attending surgeons as stable to be transferred to MRI scanning room. All examinations were performed with a 3 T MR system (Magnetom Trio; Siemens, Erlangen, Germany). Each patient received intravenous bolus injection of 25 μmol/kg of body weight of gadoxetic acid disodium (Gd-EOB-DTPA, Primovist, Bayer Schering Pharma, Berlin, Germany) at a flow rate of 2 mL/sec, followed by 10 mL saline flush. Images of CEMRC in axial plane were acquired at 10, 20, 30 and 90 minutes time points respectively using three dimensional gradient-echo (GRE) T1-weighted volume interpolated breath-hold examination (VIBE) technique. Coronal plane images of T1-weighted VIBE were acquired at 30 and 90 minutes. Besides CEMRC, axial images of T1-weighted VIBE at arterial phase, portal venous phase and equilibrium phase as well as axial T2-weighted images with and without fat saturation were also obtained. The imaging techniques was summarized in Supplementary Table S1. After CEMRC of 30 minutes time point were obtained, patients were helped getting off the examination table. While they were at MRI observation room being re-checked of fitness for delayed scan, the technologists were scanning the next scheduled patient that usually took 40 minutes to complete. Subsequently, the technologists would help our patients getting on the MRI table again to acquire CEMRC of 90 minutes time point.

### Optimization of CEMRC scanning time

The CEMRC of different acquisition times were separated into different sets of images. Each set of images was randomly given a record number without chronological order and transferred to a picture archiving and communication system for analysis. Three radiologists (13, 13, 12 years of experience in abdominal radiology respectively) who did not have access to the entire dataset were also blinded to the clinical course and final diagnosis. In a research meeting, these three radiologists were briefed on the properties of gadoxetic acid disodium, scoring scheme of biliary tree visualization and imaging features of bile leak on CEMRC.

They were asked to independently score biliary tree visualization as well as presence or absence of bile leak. The scores of biliary tree visualization was recorded on a 5-point Likert scale (1 = very poor visualization defined as no opacification of bile duct, 2 = poor visualization defined as opacification of common bile duct only, 3 = fair visualization defined as opacification of common bile duct to confluence of right and left main bile ducts, 4 = good visualization defined as opacification of common bile duct to first-order division of either right or left intrahepatic bile ducts, 5 = excellent visualization defined as opacification of common bile duct to at least second-order division of either right or left intrahepatic bile ducts). Bile leak on CEMRC was defined as extravasation of liver-excreted contrast medium from bile duct. The detection of bile leak was recorded independently on a five-point Likert scale (1 = very unlikely, 2 = unlikely, 3 = equivocal, 4 = likely, 5 = very likely).

### Final decision and characterization of bile leak on CEMRC

The final decision on whether bile leak was present or absent was determined by an expert-panel who had full knowledge of all CEMRC, available additional examinations, laboratory data and clinical information of the patients. This expert-panel comprised two abdominal radiologists and one trauma surgeon who had more than 20 years of experience in trauma radiology. None of them was involved or had influence over the scoring of biliary tree visualization and bile leak detection. All data were studied by this expert-panel group to reach a consensus of whether or not bile leak was present or absent. Characterization of the bile leak based on morphology of extravasated biliary contrast medium was further categorized by the expert-panel. The bile leak was characterized as type I if it was confined, and characterized as type II if it was expanding in the presence of liver capsule disruption.

### Records of clinical parameters, specific treatments for biliary injuries, biliary-related complications and length of hospital stay

All patients were followed up for a median of 19.8 months (IQR 10.5, 29.8 months).Their admission CT reports were reviewed for AAST grading of liver trauma and active extravasation of vascular iodinated contrast medium. Their admission notes were reviewed for serum liver enzymes and bilirubin levels on arrival as well as initial treatment for liver trauma (observation or angioembolization). Their medical records were reviewed for the choice of specific treatment for biliary injuries, biliary-related complications and the length of hospital stay.

### Statistics

Statistical analyses were computed with a software package (IBM SPSS Statistics version 21). Intraclass Correlation Coefficient (ICC) of scores on biliary tree visualization and bile leak detection were computed to estimate interrater reliability. It was categorized as poor (ICC < 0.5), fair (ICC of 0.5–0.7), good (ICC of 0.7–0.8), very good (ICC of 0.8–0.9) or excellent (ICC of 0.9–1.0). Accuracy of bile leak detection was measured by the area under receiver operating characteristic curve (AUROC). Differences of data between the groups of (1) bile leak versus no bile leak, and (2) type I versus type II bile leak were compared. Categorical data were compared using Fisher’s exact test. Continuous data were compared with Mann-Whitney test. The differences were considered significant if the 2-tailed p-values were less than 0.050.

## Electronic supplementary material

Figure S1

Table S1

## Data Availability

The datasets generated during and/or analysed during the current study are not publicly available due to institutional policy but are available from the corresponding author on reasonable request.
