# Supplementary material for: Detection and characterization of traumatic bile leaks using Gd-EOB-DTPA enhanced magnetic resonance cholangiography
Source: Sci Rep. 2018 Oct 2;8:14612. doi: 10.1038/s41598-018-32976-0 (PMC6168538; doi:10.1038/s41598-018-32976-0)

Title:

Detection and characterization of traumatic bile leaks using Gd-EOB-DTPA enhanced magnetic resonance cholangiography

Yon-Cheong Wong[1] M.D. (Corresponding author), Li-Jen Wang[1] M.D., M.P.H, Cheng-Hsien Wu[1] M.D., Huan-Wu Chen[1] M.D., Chen-Ju Fu[1] M.D., Kuo-Ching Yuan[2] M.D., Being-Chuan Lin[2] M.D., Yu-Pao Hsu[2] M.D., Shih-Ching Kang[2] M.D.

Figure S1: A type II bile leak in a 23-year-old man with a grade IV liver injury treated by angioembolization (not shown). (a) Contrast enhanced MR cholangiography acquired at 90 minutes in coronal plane shows a type II bile leak (arrow) with subhepatic space extension. Liver lacerations (arrowheads) also extend to a large defect at right lateral side of the liver resulting in a huge biloma. (b) Endoscopic retrograde cholangiography depicts a direct bile leak (arrow) from an injured intrahepatic bile duct. He was treated with sphincterotomy and endoscopic retrograde biliary drainage.

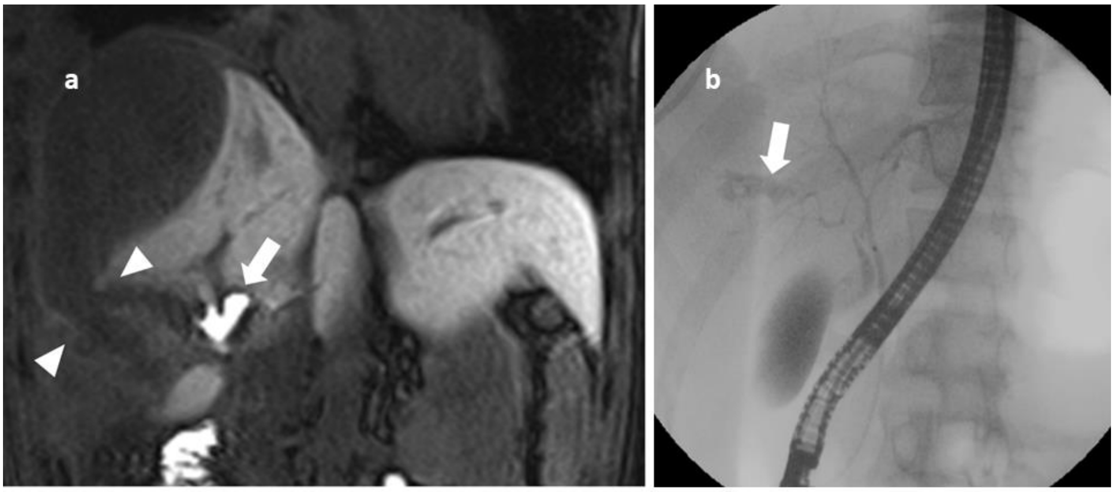

Supplement: Supplementary file 1 — Figure S1 [file 41598_2018_32976_MOESM1_ESM.pdf]
