# Supplementary material for: Detection and characterization of traumatic bile leaks using Gd-EOB-DTPA enhanced magnetic resonance cholangiography
Source: Sci Rep. 2018 Oct 2;8:14612. doi: 10.1038/s41598-018-32976-0 (PMC6168538; doi:10.1038/s41598-018-32976-0)
Supplement: Supplementary file 2 — Table S1 [file 41598_2018_32976_MOESM2_ESM.pdf]

**Title:**

Detection and characterization of traumatic bile leaks using Gd-EOB-DTPA enhanced magnetic resonance cholangiography

Yon-Cheong Wong[1] M.D. (Corresponding author), Li-Jen Wang[1] M.D., M.P.H, Cheng-Hsien Wu[1] M.D., Huan-Wu Chen[1] M.D., Chen-Ju Fu[1] M.D., Kuo-Ching Yuan[2] M.D., Being-Chuan Lin[2] M.D., Yu-Pao Hsu[2] M.D., Shih-Ching Kang[2] M.D.

Table S1. Protocol and parameters of contrast-enhanced magnetic resonance cholangiography (CEMRC). T1-weighted images were obtained with volume interpolated breath-hold examination (VIBE) and T2-weighted images were obtained with half Fourier acquisition single short turbo spin echo (HASTE) techniques.

| **Parameters** | **T1-VIBE (axial)** | **T2-HASTE (axial)** | **CEMRC (axial)** | **CEMRC (coronal)** |
|---|---|---|---|---|
| **TR/TE** | 3.32/1.17 | 1200/89 | 3.32/1.17 | 3.32/1.17 |
| **Number of averages** | 1 | 1 | 1 | 1 |
| **Flip angle** | 13 | 150 | 35 | 35 |
| **Field of view(mm)** | 350 | 330 | 350 | 350 |
| **Matrix size** | 256*80% | 320*80% | 256*80% | 256*80% |
| **Slice thickness(mm)** | 2.5 | 5 | 2.5 | 2.5 |
| **Slice gap** | 2.5 | 5 | 2.5 | 2.5 |
| **Number of slice** | 72 | 28 | 72 | 56 |
| **Acquisition plane** | Axial | Axial | Axial | Coronal |
| **Half-Fourier fact** | Off | 5/8 | Off | Off |
| **Parallel imaging acceleration factor** | 2 | 2 | 2 | 2 |
| **Receiver bandwidth(Hz/pixel)** | 500 | 781 | 500 | 500 |
| **Turbo factor** | N/A | 179 | N/A | N/A |
| **Oversampling** | 0% | 0% | 30% | 30% |
